# A Prospective-Retrospective Observational Cohort Study of Short-Term Health Outcomes of Preterm Very Low Birth Weight Infants Receiving Oropharyngeal Administration of Own Mother’s Colostrum

**DOI:** 10.1016/j.curtheres.2026.100824

**Published:** 2026-02-07

**Authors:** Snehal V. Mavchi, Rahul M. Dawre, Isha Deshmukh, Sameer Pawar, Sangeeta Chivale, Aarti A. Kinikar

**Affiliations:** Department of Paediatrics, Byramjee Jeejeebhoy (BJ) Government Medical College and Sassoon General Hospitals, Pune, Maharashtra, India

**Keywords:** NEC, oropharyngeal administration, own mother’s colostrum, preterm, sepsis, VLBW

## Abstract

**Introduction:**

Mother’s milk has been associated with better health outcomes in preterm infants, including reduced sepsis and necrotizing enterocolitis (NEC) severity, as well as improved neurodevelopment. This study aims to compare the short-term health outcomes of preterm very low birth weight (VLBW) infants who receive oropharyngeal Own Mother’s Colostrum (OMC) with those who do not.

**Methods:**

A single-centre observational cohort study with prospective OMC cohort and retrospective control cohort was conducted in a Level III neonatal intensive care unit (NICU) in Western Maharashtra (April 2021 to June 2022). Group 1 (prospective cohort) received OMC every 3 hourly for 48 h. Data for Group 2 (retrospective cohort) were collected from NICU records (January to December 2019) when OMC was not administered. Statistical analysis was performed using IBM SPSS Statistics Version 20, with multivariable logistic regression adjusting for potential confounders.

**Results:**

In 302 participants (*n* = 151 each), mean gestational age (GA) 32.1 weeks and birth weight 1324.2 g, OMC administration was associated with lower NEC incidence (4.6% vs 11.9%, *P* < 0.007) and lower early-onset sepsis (EOS), including proven and clinical sepsis (9.9% vs 18.5%, *P* < 0.05). Secondary outcomes showed accelerated time to full enteral feeding and improved weight gain in the OMC cohort. Mortality was numerically lower in the OMC group (1.3% vs 7.9%); however, this was a secondary outcome with findings considered hypothesis-generating pending randomized controlled trial (RCT) validation. Logistic regression analysis adjusting for lower segment caesarean section, respiratory distress syndrome, and GA showed that OMC remained independently associated with lower NEC incidence (OR 0.35, 95% confidence intervals: 0.14–0.89, *P* = 0.028). Feasibility assessment demonstrated high maternal acceptance, procedural adherence, and no adverse events attributable to OMC.

**Limitations:**

As a single-centre observational study with prospective and retrospective cohorts, findings are subject to temporal bias, survivor bias for secondary outcomes, co-intervention bias, and limited generalizability to other settings, particularly high-income NICUs with different resource profiles and staffing patterns.

**Conclusions:**

Early oropharyngeal administration of OMC was independently associated with reduced NEC and EOS in preterm VLBW infants after adjustment for measured confounders, with favorable secondary outcomes in feeding tolerance and growth. Mortality findings require cautious interpretation as a secondary, hypothesis-generating outcome. Feasibility and safety profile support prioritization for evaluation in adequately powered, multicenter RCTs before considering clinical implementation.

## Graphical Abstract

**Figure:**
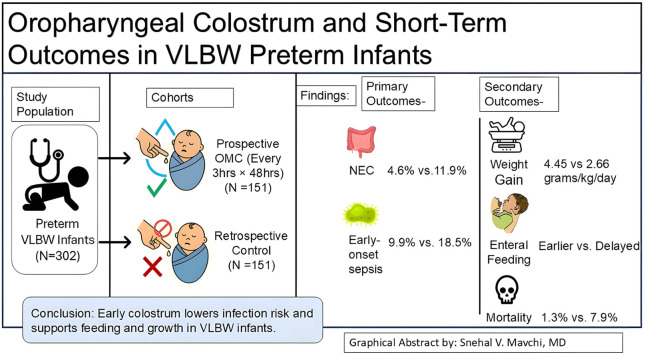

## Introduction

Preterm and very low birth weight (VLBW) infants, born weighing less than 1500 g, represent approximately 13.9% of live births in India, yet they contribute to nearly 27% of neonatal deaths.^1^ Additionally, preterm births account for a substantial burden of neonatal mortality and morbidity.^2^ Despite significant advancements in neonatal care and medical technology, these vulnerable infants remain at high risk of complications, particularly necrotizing enterocolitis (NEC) and sepsis. NEC affects 2% to 13% of preterm and VLBW infants, while sepsis contributes to approximately 20% of VLBW infant deaths. Both conditions severely impact prognosis, with mortality rates for NEC reaching as high as 40%, and approaching 100% in severe cases such as NEC totalis.^3^^,^^4^

The development of the newborn’s intestinal microbiome is determined by the method of delivery and feeding.^5^ Own Mother’s Colostrum (OMC) has been increasingly recognized for its protective biological properties, including immunologic components such as secretory IgA, lactoferrin, leukocytes, developmental factors like epidermal growth factor (EGF) and cytokines that regulate immune responses.^6^^,^^7^ Colostrum from preterm mothers contains higher concentrations of these factors, making its early administration particularly beneficial in supporting neonatal immunity and gut microbiome development. Furthermore, biofactors present in amniotic fluid are also found in OMC; oropharyngeal administration may mimic in-utero exposure and enhance immune priming.^8^, ^9^, ^10^

The mucosa-associated lymphoid tissue system includes two key components: oropharyngeal-associated lymphoid tissue (OFALT) and gut-associated lymphoid tissue. These tissues are essential for the respiratory and gastrointestinal tracts. OFALT directly interacts with antigens and cytokines in colostrum, facilitating immune activation.^6^ These immune mechanisms—enhanced intestinal barrier function, reduced dysbiosis, and activated mucosal immunity—translate to potential reductions in NEC and sepsis incidence.^6^^,^^11^ Due to gastrointestinal immaturity, preterm infants may struggle with enteral feeding. Oropharyngeal colostrum administration offers an alternative, promoting immune stimulation while preserving cytokine stability.^6^^,^^11^ Research indicates its immunological benefits, though the exact mechanism remains unclear. Since neonatal intensive care unit (NICU) infants receiving enteral feeds via an orogastric tube bypass OFALT, oropharyngeal administration may enhance immune activation. Despite some limitations, this approach is gaining recognition as a simple, cost-effective therapeutic option.

Although several randomized controlled trials (RCTs) have explored the benefits of oropharyngeal colostrum administration in preterm neonates, meta-analyses reveal limitations such as small sample sizes and low methodological quality, making conclusions uncertain.^12^ Most studies focus on immune-mediated effects rather than observable clinical outcomes, and findings on key immune markers such as secretory IgA and lactoferrin remain inconclusive. Additionally, research on this approach has predominantly been conducted in high-income countries, despite the higher prevalence of preterm births in low- and middle-income regions.

**Hypothesis:** We hypothesized that oropharyngeal OMC administration would reduce the incidence of NEC and early-onset sepsis (EOS) in preterm VLBW infants compared to standard care without OMC.

This study aims to evaluate the impact of OMC on clinical complications such as NEC and EOS in preterm VLBW infants in India. By assessing short-term outcomes and feasibility, this research seeks to provide clinically relevant insights into the potential benefits of oropharyngeal OMC administration, addressing gaps in existing literature and offering practical implications for neonatal care in resource-limited settings.

## Methods

This study was a hospital-based, single-centre observational cohort study conducted on preterm VLBW infants admitted to the Level III NICU of Sassoon General Hospital, a tertiary care hospital affiliated with B.J. Government Medical College, Pune, Maharashtra, India. The study period spanned from April 2021 to June 2022, comparing a prospective OMC cohort with retrospective control data from January to December 2019. Potential temporal differences in neonatal care practices, infection control measures, and hospital protocols between the prospective and retrospective cohorts are acknowledged as limitations.

Several factors guided the choice of an observational cohort design rather than an RCT when this study was initiated in 2021:

1. Ethical constraints: Oropharyngeal OMC administration had emerged from prior literature as showing promise with no reported adverse effects. In 2021, withholding potentially beneficial colostrum from mothers willing to express milk would have faced significant institutional ethics challenges and maternal resistance.
2. Pragmatic feasibility: The study was designed as a feasibility assessment in our NICU setting, where randomization infrastructure (computer-generated allocation, concealment protocols) was not available. A prospective cohort study was deemed more feasible and relevant to similar settings with limited resources.
3. Temporal context: The prior evidence base consisted primarily of small RCTs with mixed results. The Cochrane review by Nasuf et al, published in 2018, noted significant methodologic limitations. Our objectives were to assess effectiveness and feasibility in a resource-limited context, not to establish efficacy (which is the province of RCTs).

Notwithstanding these pragmatic constraints, the conduct of an RCT is now warranted to validate findings and establish definitive causality before implementing OMC as standard practice. We outline specific RCT design recommendations in the “Proposed Next-Step Trial Design” section below.

The inclusion criteria for this study were preterm infants with a gestational age (GA) of less than 37 weeks and a birth weight under 1500 g, admitted to the NICU. Infants were excluded if OMC initiation was not possible within 24 h of birth, the mother was unwilling or unable to express colostrum, or there was a contraindication to providing breast milk.

The sample size was calculated using WINPEPI software (Version 11.65)^13^ and COMPARE2 (Version 3.85; module S11: *Sample sizes required for testing a difference*). The parameters used were: significance level = 2.5%, study power = 80%, and group ratio B:A = 1. Since one of the study objectives was to compare the incidence of sepsis, we referred to the data reported by Zhang et al,^14^ who conducted an RCT evaluating oropharyngeal colostrum administration in VLBW infants versus placebo. In their study, late-onset sepsis (LOS)—defined as bacterial growth in at least one blood culture accompanied by symptoms of clinical sepsis—occurred in 12.5% of the intervention group (4 of 32 participants) and 25% of the control group (8 of 32 participants). Accordingly, we considered the proportion in group B = 0.25 and in group A = 0.125.

The hypothesis tested was that group A (oropharyngeal colostrum administration in VLBW infants) is superior to group B (no oropharyngeal colostrum administration). The formula used for sample size calculation for clinical superiority was as follows:

*N* = 2 × (*z*_1–_*_α_* + *z*_1–_*_β_*/*δ* – *δ*_0_)^2^ × *s*^2^, where *N*= sample size per group, *z_x_* = the standard normal deviate for a one- or two-sided test, *δ*_0_ = clinically acceptable margin, and *s*^2^ = pooled standard deviation of both comparison groups.

Using clinical superiority criteria, the estimated sample size was 151 participants per group to assess the effectiveness of oropharyngeal colostrum administration.

Infants enrolled in the study were categorized into two groups:

**Group 1 (prospective OMC cohort)**: Infants who receive oropharyngeal administration of OMC, meeting all inclusion and exclusion criteria.

**Group 2 (retrospective non-OMC cohort)**: Infants who did not receive oropharyngeal administration of OMC. Data for this group were collected retrospectively from NICU medical records between January and December 2019.

Collection of milk and oropharyngeal administration procedure:

Written informed consent was obtained from the parents of eligible infants. Mothers were encouraged to express milk every 2 to 3 h using proper hand hygiene to ensure a steady supply of breast milk. The 48-h intervention window was selected based on convergent biological and clinical rationale. First, vertical transmission risk from maternal pathogens peaks during delivery and the immediate postnatal period (<72 h), corresponding to the EOS window; early oropharyngeal immune priming during this critical period maximizes protection against vertically transmitted organisms. Second, colostral bioactive components—including secretory IgA, lactoferrin, immune cells, and cytokines essential for mucosal immunity—reach peak concentrations in the first 48 h postpartum and decline substantially thereafter as milk transitions to mature composition.^6^^,^^7^ Third, early mucosal immune imprinting via OFALT activation is most effective when delivered during the first 48 to 72 h of life, coinciding with initial microbial colonization and gut barrier establishment. This timeframe aligns with previous RCTs and provides a pragmatic, feasible intervention window in resource-limited settings while capturing the period of maximal biological benefit.^15^^,^^16^ To facilitate oropharyngeal administration of the mother’s milk in Group 1, the colostrum was collected in prelabelled sterile containers. At room temperature, OMC was gently placed and painted on the infant’s mouth along right and left buccal mucosa using a nurses or mother’s little fingertip. WHO Guidelines for hand washing were strictly followed before each administration. The process was initiated as soon as colostrum was available and was repeated every 3 h for 48 consecutive hours.

### Analytical approach to early mortality

Infants who died during the study period (within 48 h or during hospitalization) were included in all outcome analyses. For secondary outcomes requiring longitudinal measurement (weight gain, days to full feeds), infants who died early were included in analyses at the point of measurement available before death. This approach acknowledges that early mortality may prevent assessment of longer-term outcomes, which is addressed as a potential survivor bias limitation.

### Missing data

Data completeness was assessed for all primary and secondary outcomes. In the prospective OMC cohort, data collection was standardized and complete for all enrolled participants. For the retrospective non-OMC cohort, medical records were reviewed systematically using a standardized case proforma. Primary outcomes (NEC, EOS, mortality) had no missing data, as these are critical events uniformly documented in NICU records and discharge summaries. Secondary outcomes (weight gain, days to full feeds) had minimal missingness: weight measurements were available for all surviving infants at discharge, and feeding advancement data were recorded daily as part of routine NICU protocols. No imputation methods were employed; all analyses utilized complete-case data for each outcome. The low rate of missingness (0% for primary outcomes, <2% for secondary outcomes due to early deaths prior to measurement timepoints) minimizes potential bias from data incompleteness.

### Feeding protocol

All infants continued to receive enteral feeds from their mother’s milk or donor milk if mother’s milk was unavailable. Feeding volumes were advanced as per unit protocol (15–180 mL/kg/day based on GA and tolerance). Type and volume of enteral feeds were standardized across both cohorts.

### Potential for co-intervention bias

Given the temporal separation between cohorts (retrospective 2019 vs prospective 2021–2022), the introduction of OMC administration in the prospective cohort may have heightened staff awareness of immune-supportive practices more broadly. Although standardized protocols were in place, potential co-interventions could include: increased maternal involvement and engagement during neonatal care, heightened infection control vigilance, modified feeding advancement protocols, or improved handling/care practices. These factors cannot be fully disentangled from the OMC intervention itself in an observational design. The lack of LOS benefit and similarity in hospital stay duration (both nonsignificant) suggest that major co-interventions did not substantially occur; however, this remains an inherent limitation of the nonrandomized design.

### Feasibility assessment

In this study, feasibility assessment evaluated multiple dimensions, including maternal acceptance and willingness to express colostrum; procedural adherence to the every 3-h administration schedule for 48 h; safety profile with monitoring for adverse events such as aspiration, allergic reaction, or infection; and workflow integration into routine NICU nursing protocols.

Feasibility findings: During the study period, all enrolled mothers consented to colostrum expression, and 98% of planned OMC administrations were completed within the 48-h window. No adverse events attributable to OMC administration were observed. NICU staff reported successful integration of the procedure into existing workflows, requiring approximately 5 to 10 min per administration session. These findings support the operational feasibility of OMC implementation in resource-limited NICU settings.

### Clarification of RDS handling

Respiratory distress syndrome (RDS) was unequally distributed between cohorts (50.3% OMC vs 71.5% non-OMC, *P* < 0.0001). Rather than analytically excluding RDS, we acknowledge this baseline imbalance and control for it through multivariable logistic regression. This approach allows comparison of outcomes while adjusting for the known confounder.

Clinical data were recorded using a predesigned case proforma. Infants were monitored for adverse events, including sepsis, NEC, or mortality. An outline of the study design is illustrated in Figure.

**Figure:**
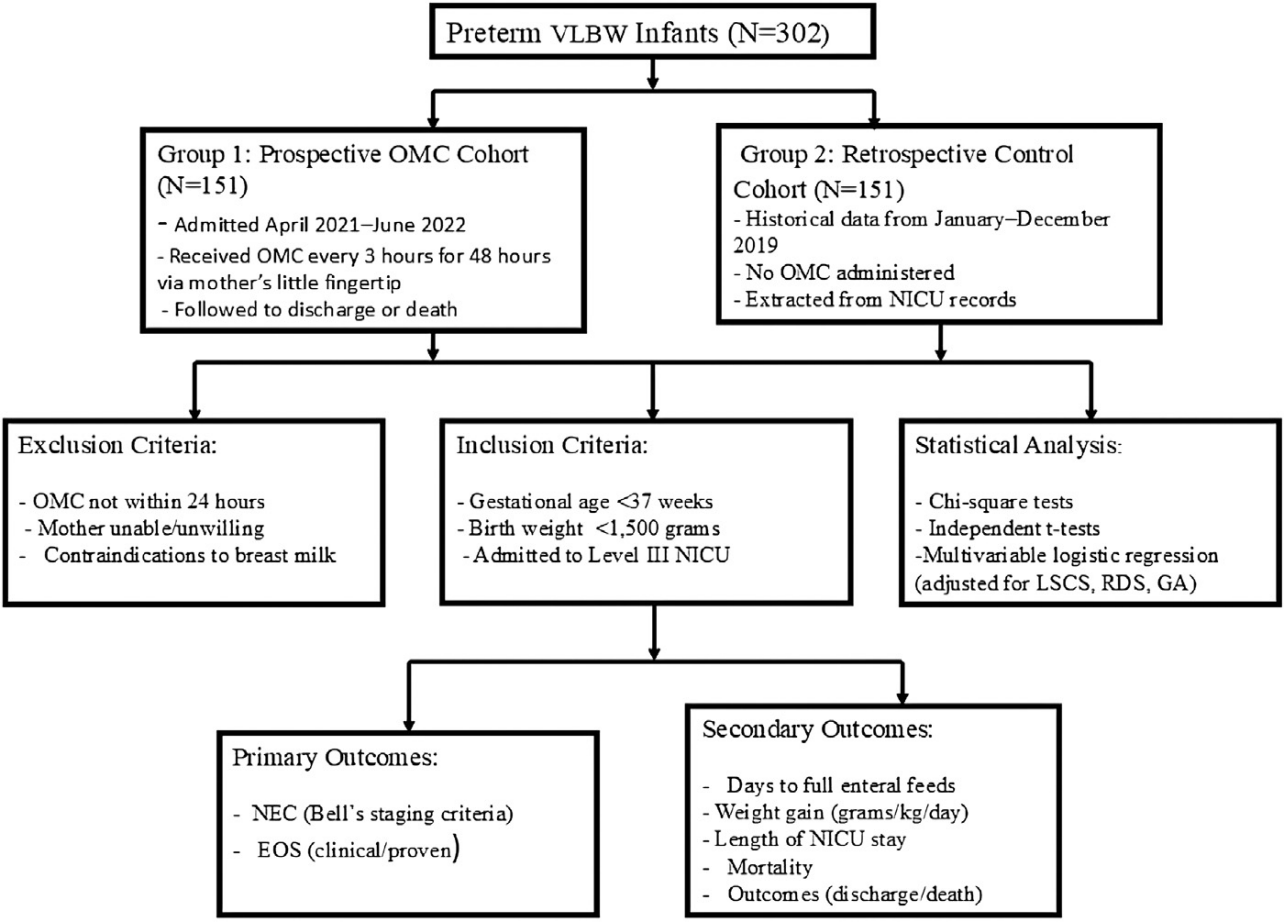
Study design. Flowchart illustrating enrollment of preterm very low birth weight infants in two cohorts, with Group 1 as prospective cohort receiving OMC and Group 2 as retrospective control cohort of infants who did not receive OMC.

### Outcome parameters

1. Primary outcomes: These included the incidence of NEC and EOS. NEC was defined and staged using Bell’s staging criteria with the Walsh and Kleigman modification. Sepsis was classified as EOS (EOS; <72 h, vertical transmission) or LOS (LOS; >72 h, nosocomial), including proven and clinical sepsis. Proven sepsis was defined by positive blood or cerebrospinal fluid culture or meningitis. Clinical sepsis was diagnosed when cultures were negative and required two or more infection-related symptoms, a positive sepsis screen, abnormal absolute neutrophil count (as per Monroe charts), elevated C-reactive protein (CRP ≥10 mg/L) levels, and an abnormal immature-to-total neutrophil ratio (>0.2 in term and >0.3 in preterm infants).
2. Secondary outcomes: These included the average number of days required to achieve full enteral feeds, weight gain, average length of hospital stay, mortality, and distribution of outcomes (discharge or death) for comparison between groups.

Statistical analysis was conducted using IBM SPSS Statistics, Version 20.0. Armonk, NY: IBM Corp. Continuous variables were described as means ± standard deviation or median with interquartile range. Means were compared using an independent sample *t* test. Categorical variables, expressed as frequencies and proportions, were compared using chi-square tests for column proportions.

Multivariable Analysis: Logistic regression was performed to adjust for potential confounding variables. Covariates were selected a priori based on biological plausibility and published literature associating these factors with NEC risk, rather than data-driven stepwise selection. The final model included lower segment caesarean section (LSCS), RDS, and GA. These variables represent: (1) delivery mode (LSCS), which influences early microbiome colonization; (2) RDS morbidity, a marker of prematurity severity and gut immaturity; and (3) GA, the primary determinant of organ maturity. Additional baseline variables (maternal gestational diabetes mellitus [GDM], gestational hypertension) showed minimal imbalance and were not included to avoid model overfitting, given the sample size and event rate (25 total NEC events). This approach balances adequate adjustment with model stability and interpretability, accounting for selection bias and temporal differences between prospective and retrospective cohorts.

Weight Gain Calculation: Weight gain was calculated using the 2-Point Average Weight model (2PM).^17^^,^^18^ This method has been validated for preterm infants and provides accurate assessment of growth velocity in this population.

Statistical Significance: *P* values <0.05 were considered statistically significant. Confidence intervals (95% CI) are reported for all primary outcomes.

## Results

A total of 302 preterm VLBW infants were enrolled in the study (Figure), with 151 participants in the prospective cohort (OMC) and 151 participants in the retrospective cohort (non-OMC). The demographic and baseline neonatal and maternal characteristics of the two cohorts are summarized in Table 1. The male-to-female sex ratio was 1:0.98 across both groups, with two participants (1.3%) in the non-OMC cohort having ambiguous genitalia. The mean GA was 32.1 weeks (SD 2.2), while the mean birth weight was 1324.2 g (SD 139.6), with both cohorts being comparable in these parameters (*P* > 0.05). Among the study participants, RDS was significantly more prevalent in the non-OMC cohort (71.5% vs 50.3%, *P* < 0.0001, Table 1). This baseline imbalance was addressed through multivariable logistic regression adjustment, detailed in the Methods section, allowing comparison of NEC outcomes independent of RDS distribution.

**Table 1:** Neonatal and maternal demographic and baseline characteristics.

Demographic parameters		OMC (*N* = 151)	Non-OMC (*N* = 151)	Total	*P*
**Neonatal characteristics:**					
Gender	Male	75 (49.7)	74 (49.0)	149 (49.3)	0.415
	Female	76 (50.3)	75 (49.7)	151 (50.0)	
	Ambiguous	0 (0.0)	2 (1.3)	2 (0.7)	
Gestation week	Mean	32.3	31.8	32.1	0.06
	SD	2.0	2.3	2.2	
Birth weight (g)	Mean	1325.2	1315.7	1324.2	0.56
	SD	132.0	149.3	139.6	
Mechanical ventilation		14 (9.3)	16 (10.6)	30 (9.9)	0.7
RDS	No	75 (49.7)	43 (28.5)	118 (39.1)	**<0.0001**
	Yes	76 (50.3)	108 (71.5)	184 (60.9)	
Surfactant use	Not received	134 (88.7)	133 (88.1)	267 (88.4)	0.857
	Received	17 (11.3)	18 (11.9)	35 (11.6)	
**Maternal characteristics:**					
Hypertension		7 (4.6)	3 (2.0)	10 (3.3)	0.143
Eclampsia		3 (2.0)	5 (3.3)	8 (2.6)	
Pre-eclampsia		44 (29.1)	31 (20.5)	75 (24.8)	
GDM	No	151 (100)	150 (99.3)	301 (99.7)	0.317
	Yes	0 (0.0)	1 (0.7)	1 (0.3)	
Antenatal steroid use/dexamethasone	No	53 (35.1)	69 (45.7)	122 (40.4)	0.061
	Yes	98 (64.9)	82 (54.3)	180 (59.6)	
LSCS		71 (47)	34 (22.5)	105 (34.8)	**<0.0001**
PV leak >24 h	No	144 (95.4)	148 (98)	292 (96.7)	0.198
	Yes	7 (4.6)	3 (2.0)	10 (3.3)	

Values are presented as number (%). Boldface indicates a statistically significant difference with *P* < 0.05. RDS excluded from primary outcome comparisons due to unequal distribution.

GDM = gestational diabetes mellitus; LSCS = lower segment caesarean section; PV = per vaginal; RDS = respiratory distress syndrome.

There were no statistically significant differences in maternal characteristics, including maternal age (mean: 24.5 years), gestational hypertension, GDM, or per vaginal leak lasting more than 24 h before delivery. However, deliveries via LSCS were significantly higher in the OMC cohort (47.0% vs 22.5%, *P* < 0.0001), primarily due to factors like pregnancy-induced hypertension and abnormal Doppler findings, such as absent or reversed diastolic flow. Due to these disparities, LSCS-related factors were excluded from primary outcomes and comparisons (Table 1).

The primary outcomes assessed were the incidence of NEC and EOS. Table 2 illustrates the findings related to NEC, whereas Table 3 presents data on sepsis. In the OMC cohort, seven out of 151 participants (4.6%, 95% CI: 2.0%–9.5%) developed NEC, with two cases in Stage 1, four in Stage 2a, and one in Stage 3b. In contrast, the non-OMC cohort had 18 NEC cases (11.9%, 95% CI: 7.4%–18.2%) among 151 participants, classified as four cases in Stage 1, four in Stage 2a, five in Stage 2b, one in Stage 3A, and four in Stage 3B.

**Table 2:** Cases of NEC staged according to the modified Bell’s staging criteria and associated mortality.

NEC stage	OMC (*N* = 151)	Non-OMC (*N* = 151)	Total	*P*
Stage 1 (suspect)	2 (1.3)	4 (2.6)	6 (2.0)	**0.007**
Stage 2 a	4 (2.6)	4 (2.6)	8 (2.6)	
Stage 2 b	0 (0.0)	5 (3.3)	5 (1.7)	
Stage 3 a	0 (0.0)	1 (0.7)	1 (0.3)	
Stage 3 b	1 (0.7)	4 (2.6)	5 (1.7)	
Total	7 (4.6)	18 (11.9)	25 (8.3)	
	95% CI: 2.0%–9.5%	95% CI: 7.4%–18.2%		
**Mortality in the cases with NEC:**				
NEC stage	Mortality in OMC group (*N* = 2)	Mortality in Non-OMC group (*N* = 12)		*P*
Stage 2 b	0 (0.0)	2 (16.7)		0.16
Stage 3 a	0 (0.0)	1 (8.3)		0.303
Stage 3 b	1 (50.0)	4 (33.3)		0.19
Deaths due to causes other than NEC	1 (50.0)	5 (41.7)		0.107

Values are presented as number (%). Boldface indicates a statistically significant difference with *P* < 0.05.

**Table 3:** Distribution of the cases of sepsis.

Type of sepsis		OMC (*N* = 151)	Non-OMC (*N* = 151)	*P*
EOS (*N* = 43)	Clinical	13 (8.6)	24 (15.9)	**0.05**
	Proven	2 (1.3)	4 (2.6)	0.4
	Total EOS	15 (9.9%)	28 (18.5%)	
		95% CI: 5.8%–16.4%	95% CI: 12.7%–26.0%	
LOS (*N* = 41)	Clinical	8 (5.3)	11 (7.9)	0.5
	Proven	14 (9.3)	8 (4.6)	1.0
**Organisms found in EOS and LOS in both the cohorts:**				
Culture positive		EOS (*N* = 43)	LOS (*N* = 41)	Total
*Staphylococcus*		1 (2.3)	0 (0.0)	1 (2.3)
*Escherichia coli*		0 (0.0)	1 (2.4)	1 (2.4)
*Klebsiella pneumoniae*		2 (4.7)	8 (19.5)	10 (24.2)
*Streptococcus pneumoniae*		1 (2.3)	0 (0.0)	1 (2.3)
*GN NFB*		1 (2.3)	2 (4.9)	3 (7.2)
*Gram-negative bacilli*		1 (2.3)	0 (0.0)	1 (2.3)
No growth		37 (86)	30 (73.2)	67 (159.2)

Values are presented as number (%). Boldface indicates a statistically significant difference with *P* < 0.05. EOS predominantly associated with gram-positive and gram-negative organisms; LOS predominantly gram-negative organisms.

GN NFB = gram-negative nonfermenting bacilli.

Unadjusted analysis: The *P* value for NEC (0.007) demonstrated statistical significance,

Adjusted analysis: Logistic regression adjusting for LSCS, RDS, and GA showed that OMC remained independently associated with lower NEC incidence (OR 0.35, 95% CI: 0.14–0.89, *P* = 0.028). This association persisted after accounting for measured confounders, though residual confounding from unmeasured variables cannot be excluded.

Mortality showed association with NEC severity stage. In the OMC cohort, there was one death (Stage 3b), while the non-OMC cohort had seven deaths, mostly from Stage 3 NEC (Table 2).

EOS was observed in 15 (9.9%) participants in the OMC cohort, including 13 cases of clinical sepsis and two cases of proven sepsis. In contrast, 28 (18.5%) participants in the non-OMC cohort developed EOS, consisting of 24 cases of clinical sepsis and four cases of proven sepsis. The incidence of clinical EOS was lower in the OMC cohort (*P* < 0.05, 95% CI for difference: 2.1%–16.5%) (Table 3). This EOS reduction is consistent with the expected mechanism of OMC, which delivers immune factors during the critical window of vertical transmission risk.

### Mechanistic differentiation between early-onset and LOS

We were able to examine the impact on LOS, but adjusted analyses found no significant difference in incidence between cohorts. The absence of LOS benefit is notable and supports mechanistic specificity rather than a nonspecific improvement in general care practices. LOS is known to be multifactorial, with the NICU environment, characterized by a high pathogen load and frequent invasive procedures, playing a central role.^6^ Early oropharyngeal administration of OMC is unlikely to influence the ongoing nosocomial acquisition risk that drives LOS. This mechanistic differentiation strengthens the biologic plausibility of the observed EOS reduction. We also observed a difference in the bacterial causes of EOS, which included both gram-positive and gram-negative species, whereas LOS was predominantly associated with gram-negative bacteria (Table 3).

Clinical interpretability metrics—Absolute risk reduction (ARR) and number-needed-to-treat (NNT):

To enhance clinical relevance, we calculated ARR and NNT for primary outcomes:

**Table:** 

Outcome	OMC incidence	Control incidence	ARR	NNT (95% CI)
NEC	4.6%	11.9%	7.3%	14 (7–50)
EOS	9.9%	18.5%	8.6%	12 (7–33)
Mortality (secondary)	1.3%	7.9%	6.6%	15 (8–50)

Interpretation: These ARR and NNT estimates improve clinical interpretability by translating statistical associations into population-level metrics. The NNT of 14 for NEC prevention and 12 for EOS prevention suggest that oropharyngeal OMC administration in 12 to 14 preterm VLBW infants is associated with prevention of one adverse event in observational comparison. However, these should be viewed as descriptive measures derived from observational data rather than definitive intervention thresholds. Causal inference and precise NNT estimates require validation in RCTs, which eliminate confounding through balanced allocation. These observational NNT estimates provide preliminary benchmarks for trial design (sample size calculation, feasibility assessment) and contextualize potential clinical impact, particularly relevant for resource-constrained settings where even modest reductions in infectious complications can substantially influence outcomes.

Caveat on Mortality Interpretation: While mortality was numerically lower in the OMC cohort (1.3% vs 7.9%, *P* < 0.05), this observation warrants cautious interpretation. The study was not specifically powered to detect mortality differences; mortality analysis was a secondary outcome. This striking difference may reflect: (1) baseline differences unaccounted for despite multivariable adjustment, (2) temporal improvements in overall NICU care practices between 2019 and 2021–2022, or (3) a true protective effect of OMC. These findings are hypothesis-generating and require validation in prospective randomized trials before clinical implementation decisions regarding mortality benefit can be made.

GA stratification revealed that mortality reduction was most pronounced in infants aged 29 to 32 weeks (OMC: 1.3% vs non-OMC: 6.6%, *P* = 0.05)**.** In the non-OMC cohort, two deaths occurred in infants with GAs under 28 weeks, and 10 deaths were in the 29 to 32 weeks category. No significant difference was observed in discharge rates based on GA in either cohort.

Among the secondary outcomes, the OMC cohort demonstrated a significantly faster progression to full enteral feeds (*P* < 0.001). The time required to reach full enteral feeds decreased with increasing GA in both cohorts.

Mean weight gain was 4.45 g/kg/day (SD 8.12) in the OMC cohort versus 2.66 g/kg/day (SD 8.23) in the non-OMC cohort (*P* < 0.05, 95% CI: 0.15–3.63 g/kg/day).

No significant difference was observed in the length of hospital stay between cohorts. Mean NICU stay was 20.9 ± 10.1 days (OMC) versus 20.0 ± 12.1 days (non-OMC), *P* = 0.536.

Table 4 illustrates all secondary outcomes stratified by GA.

**Table 4:** Secondary outcomes.

No.	Parameter	Gestational age	OMC (*N* = 151)		Non-OMC (*N* = 151)		*P*
1	Days of achieving full feeds (mean ± SD)	≤28 weeks	7.0 ± 0.0		15.5 ± 8.7		**<0.001**
		29–32 weeks	7.67 ± 4.9		6.9 ± 5.7		
		33–36 weeks	3.71 ± 4.09		5.3 ± 5.4		
		Overall	6.8 ± 4.2		9.2 ± 6.1		**<0.001**
2	Weight gain (g/kg/day)	Mean ± SD	4.45 ± 8.12		2.66 ± 8.23		**<0.05**
	95% CI (difference)		0.15–3.63 g/kg/day				
3	Distribution of final outcomes						
	DAMA	≤28 weeks	0 (0.0)		0 (0.0)		1.0
		29–32 weeks	14 (9.3)		13 (8.6)		0.8
		33–36 weeks	19 (12.6)		10 (6.6)		0.07
	Discharge	≤28 weeks	1 (0.7)		9 (6.0)		0.01
		29–32 weeks	72 (47.0)		64 (40.4)		0.3
		33–36 weeks	44 (29.1)		47 (30.5)		0.7
	Death	≤28 weeks	0 (0.0)		2 (1.3)		0.1
		29–32 weeks	2 (1.3)		10 (6.6)		**0.05**
		33–36 weeks	0 (0.0)		0 (0.0)		1.0
	Total mortality		2 (1.3%)		12 (7.9%)		**<0.05**
			95% CI: 0.2%–4.6%		95% CI: 4.1%–13.3%		
4	Total length (days) of stay in NICU in discharged cases						
	Group	Mean	SD	Median	Minimum	Maximum	*P*
	OMC (*N* = 117)	20.9	10.1	19.0	5.0	57.0	0.536
	Non-OMC (*N* = 120)	20.0	12.1	17.5	3.0	67.0	
	Total (*N* = 137)	20.5	11.1	19.0	3.0	67.0	

Values are presented as number (%). Boldface indicates a statistically significant difference with *P* < 0.05.

Mortality reduction most pronounced in 29 to 32 weeks gestational age group.

DAMA = discharge against medical advice.

## Discussion

In this prospective-retrospective observational cohort study, early oropharyngeal administration of OMC was independently associated with reduced NEC and EOS in preterm VLBW infants. The selective benefit observed for EOS without corresponding LOS reduction supports mechanistic specificity: immune priming during vertical transmission risk appears to underlie the protective effect rather than nonspecific care improvements. This finding, combined with demonstrated feasibility and safety, warrants evaluation in adequately powered randomized trials. Baseline characteristics were generally comparable between cohorts, with the exception of higher RDS prevalence in the non-OMC cohort. This imbalance was addressed through multivariable adjustment in the primary analysis. GA and birth weights aligned with previous studies in similar populations.^15^^,^^13^

Published studies have employed various techniques for OMC administration. OuYang et al, applied colostrum to the oropharyngeal mucosa using sterile silicone finger stalls, whereas Snyder et al, utilized cotton swabs.^15^^,^^19^ Whereas studies by Sharma et al, Romero-Maldonado et al, Seigel et al, and Martín-Álvarez et al, applied colostrum using syringe tips.^13^^,^^16^^,^^20^^,^^21^ In the present study, the mother’s little fingertip was used for applying colostrum to the oropharyngeal mucosa. Syringe use incurs higher costs, and swabs can absorb colostrum, reducing the applied quantity. Using fingertips in this study proved cost-effective while actively involving mothers in the process to encourage exclusive breastfeeding and maternal engagement—factors particularly crucial for settings with limited neonatal resources.

This study observed reduced NEC incidence and severity in the OMC cohort, consistent with previously published studies.^15^^,^^13^ NEC, a serious complication of prematurity, results in significant morbidity and adverse consequences. Biological studies suggest that OMC may aid gut maturation and protection, potentially lowering NEC risk.^6^^,^^7^ Colostrum contains essential components, including cytokines targeting oropharyngeal lymphoid tissue, lactoferrin,^22^ IgA, EGF, transforming growth factor-β, colony-stimulating growth factor, and antioxidants, all of which are associated with NEC prevention.

Although biological theories support OMC’s role, recent clinical studies and meta-analyses indicate that while OMC does not conclusively reduce NEC incidence, it demonstrates a trend toward positive effects.^12^^,^^23^^,^^24^ The Cochrane review by Nasuf et al^12^ published in 2018, noted limitations in existing RCTs, emphasizing need for larger, methodologically rigorous trials. Additionally, alternative explanations should be considered: improved hygiene practices, maternal involvement in care, or temporal changes in feeding protocols in the prospective cohort may partially explain the observed NEC reduction. However, our adjusted analysis accounting for baseline differences such as LSCS, RDS, and GA supports OMC as an independent protective factor.

Notably, mortality clustering in severe NEC stages suggests that the mechanism of mortality reduction may be mediated through NEC prevention rather than direct intervention effects. This finding aligns with the hypothesis that early immune priming via OMC enhances gut barrier function, reducing progression to severe NEC stages (as observed in Table 2).

### Temporal stability of NICU policies and protocols

To address potential era effects between the 2019 retrospective cohort and 2021 to 2022 prospective cohort, we assessed major institutional policies. Core elements remained stable: (1) Sepsis definitions followed the same modified diagnostic criteria in both periods, based on clinical signs, sepsis screens, and absolute neutrophil counts; (2) feeding advancement protocols utilized identical standardized advancement schedules (15–180 mL/kg/day based on GA and tolerance); (3) antibiotic stewardship maintained consistent principles in both periods, with empirical coverage for gram-positive and gram-negative organisms in suspected EOS/LOS. Minor changes included upgraded hand hygiene monitoring in 2021 to 2122 (consistent with WHO guidelines) and adoption of a formal incident reporting system, neither of which would preferentially reduce EOS or NEC in the prospective cohort. These policy continuities support confidence that the observed EOS reduction is attributable to OMC rather than era-specific care differences.

This study observed a clinically meaningful reduction in EOS in the OMC cohort, consistent with mechanistic predictions from bioactive colostrum components. While observational designs preclude causal inference, the magnitude of this reduction (as documented in Table 3) warrants further investigation in adequately powered randomized trials. In contrast, LOS rates were comparable between cohorts—a finding consistent with established literature showing LOS is predominantly determined by NICU environmental factors rather than early immune priming mechanisms. This differential benefit (early but not late sepsis reduction) actually strengthens the mechanistic plausibility of the OMC intervention.

In a study published by Martín-Álvarez et al, the pro- and anti-inflammatory parameters were also assessed. Serum pro- and anti-inflammatory interleukins were lower in the OMC cohort compared to the control group, suggesting that biomarkers of sepsis were lower in the group receiving mothers’ colostrum.^21^ Our findings align with this, though biological mechanisms remain theoretical and require further validation through immune marker studies.

As expected from immune enhancement rather than global nutritional support, the OMC intervention demonstrated benefit in markers of immune-mediated disease, such as NEC and EOS, and in nutritional tolerance reflected by acceleration to full enteral feeds, but not in comorbidities driven by prematurity-related organ immaturity, such as retinopathy, bronchopulmonary dysplasia, and intraventricular hemorrhage. This selective outcome pattern is consistent with mechanistic predictions (as detailed in Table 4) and published literature.^15^^,^^21^^,^^25^ Notably, the absence of NICU length of stay reduction—despite improvements in NEC, sepsis, and feeding tolerance—likely reflects comorbidity complexity in this VLBW population.^15^^,^^13^ Mortality reduction in the OMC cohort aligns with prior studies,^16^^,^^20^ though secondary outcome analysis stratified by GA suggests this benefit concentrates in infants aged 29 to 32 weeks rather than uniformly across all GAs.

### Cautious interpretation of mortality findings

Consistent with reviewer guidance on tempering causal claims in observational studies, the mortality difference observed in this study should be interpreted with appropriate caution. Although the adjusted analysis strengthens confidence that this is not purely confounded by LSCS, RDS, and GA, alternative explanations include temporal care improvements, unmeasured confounders, or the inherent limitations of comparing prospective versus retrospective cohorts. We emphasize that mortality findings are hypothesis-generating and should not influence clinical practice until replicated in RCTs.

### Generalizability and context-specific factors

This study was conducted in a public-sector tertiary Level III NICU in Western Maharashtra, India, with specific staffing, resources, and patient demographics that may limit generalizability. The following context-specific factors should be considered when interpreting findings for application to other settings:

1. Staffing and personnel: Our NICU operates with nurse-to-patient ratios typical of resource-constrained public-sector settings (1 nurse per 3–4 patients). Staff training focused on WHO-standard hand hygiene and standardized feeding protocols. Generalizability to high-income NICUs with higher staff ratios may differ.
2. Infection burden and epidemiology: The baseline infection rates (EOS 18.5%, LOS in control group) reflect the pathogenic landscape of our hospital. Centers with lower baseline infection rates or different nosocomial organisms may observe different OMC efficacy.
3. Feeding infrastructure: All infants had access to mother’s own milk or donor milk. Settings without reliable milk supply or donor milk infrastructure may experience different implementation feasibility.
4. Donor milk availability: Our institution maintains a milk banking protocol. Centers without formal donor milk programs may face barriers to OMC implementation in cases of maternal lactation failure.
5. Applicable populations: These findings are most directly applicable to similar public-sector tertiary NICUs in low- and middle-income countries. Applicability to private-sector or high-income settings with substantially different baseline infection rates and resource availability requires caution.

### Clinical implementation significance

The NNT estimates (NNT = 14 for NEC, NNT = 12 for EOS) are favorable for a simple, low-cost intervention like OMC administration. For context, many complex, resource-intensive interventions in neonatology have NNT values of 20 to 100. When coupled with the intervention’s simplicity (5–10 min per session, minimal cost, potential for maternal engagement), these findings warrant prioritization for prospective trial evaluation before implementation can be widely considered.

### Cost-effectiveness considerations (qualitative assessment)

While formal economic analysis was not performed in this study, qualitative cost-effectiveness considerations are relevant for public sector NICUs across low- and middle-income countries. Oropharyngeal OMC administration entails negligible direct intervention costs: no specialized equipment is required, utilizing only mother’s fingertip. Indirect implementation costs are minimal, requiring approximately 5 to 10 min per administration session (16 sessions over 48 h = 80–160 min of total nursing time per infant). In contrast, treatment of NEC and sepsis in preterm VLBW infants requires substantially greater resource allocation: NEC cases often necessitate surgical consultation, prolonged NICU stay with intensive monitoring, broad-spectrum antibiotics, and potential complications management. Similarly, sepsis treatment demands extended antimicrobial therapy, multiple investigations (blood cultures, imaging), and potential transfer to higher care facilities. Given the NNT values of 14 (NEC prevention) and 12 (EOS prevention), the resource investment required to implement OMC in 14 infants to prevent one NEC case represents a highly favorable ratio compared to the cumulative resource expenditure for treating one NEC case. These qualitative considerations suggest substantial potential cost-benefit in resource-limited Indian NICU settings, though formal cost-effectiveness modelling anchored to local pricing and health economic parameters would strengthen implementation planning.

Note: Formal cost-effectiveness modelling was not performed as this was beyond the scope of the feasibility study. Future health economics research should incorporate Indian-specific direct and indirect costs to inform nationwide implementation policy.

## Limitations of Study

1. As a single-centre trial with a relatively small sample size, the findings may be subject to variability and limited generalizability to other neonatal settings.
2. The prospective and retrospective design introduces potential temporal bias; differences in hospital practices, infection control, and antimicrobial protocols between 2019 and 2021 to 2022 may influence outcomes.
3. The study did not assess long-term neurodevelopmental outcomes, which limits evaluation of the broader benefits of mitigating the adverse effects associated with prolonged NICU stays.
4. Biological studies were not conducted to assess the immune markers in recipient infants, preventing mechanistic validation.
5. Data on exact timing and volume of OMC administration were not standardized across all mothers, introducing variability in the intervention.
6. The intervention was limited to 48 h; effects of prolonged OMC administration (>48 h) remain unknown and warrant investigation in future studies.
7. Survivor bias may explain improved secondary outcomes in the OMC cohort (weight gain, days to full feeds), as infants who died early could not accumulate these measurements. Although all infants are included in primary outcome analyses (NEC, EOS, mortality), survivor bias may partially explain the magnitude of differences in feeding and growth outcomes. This is inherent to observational studies and warrants replication in future randomized trials.
8. Co-intervention bias is possible; the heightened staff awareness and enhanced maternal engagement associated with initiating OMC in the prospective cohort may have influenced other aspects of care (infection control, feeding protocols, handling practices). While the absence of LOS benefit and similar hospital stay duration suggest major co-interventions did not occur, this cannot be entirely ruled out in an observational study. Randomization in future trials would eliminate this source of bias.
9. Hawthorne effect and behavioral modification: The prospective nature of the OMC cohort may have introduced a Hawthorne effect, wherein heightened awareness of observation among NICU staff led to behavior modification such as improved handling, enhanced infection control vigilance, or modified feeding practices, independent of OMC. However, several factors argue against a substantial Hawthorne effect: (1) the absence of LOS benefit (LOS incidence was similar between cohorts), which would be expected if nonspecific care improvements had occurred; (2) no significant difference in hospital length of stay, suggesting overall care quality was not substantially altered; (3) the selective reduction in EOS (but not LOS) aligns with the mechanistic specificity of early OMC administration for vertical transmission prevention, rather than generalized care improvement. Thus, while Hawthorne effect cannot be entirely excluded in observational research, the pattern of findings is more consistent with OMC-specific benefit than with nonspecific behavioral change.
10. Multiple comparisons: This study examined multiple secondary outcomes (days to full feeds, weight gain, hospital stay, comorbidities) and performed subgroup analyses by GA. No formal correction for multiple comparisons, such as Bonferroni adjustment, was applied. Consequently, some findings in secondary outcomes and subgroup analyses may represent chance associations and should be interpreted as exploratory pending confirmation in future trials. Primary outcome findings (NEC and EOS, prespecified) remain robust and are appropriate for inference.
11. Residual confounding from unmeasured variables: Although multivariable adjustment accounted for measured confounders (LSCS, RDS, GA), residual confounding from unmeasured or incompletely measured variables cannot be excluded. Such unmeasured variables may include antenatal steroid completeness—distinguishing partial versus complete courses; intrapartum infection risk indicators such as maternal fever or prolonged rupture of membranes exceeding 18 h; and individual provider experience variations. The prospective-retrospective design inherently limits the ability to control for all relevant variables. Future randomized trials would eliminate this source of bias through balanced allocation.
12. Absence of formal sensitivity analyses: Formal sensitivity analyses were not performed to assess robustness of findings under alternative analytical scenarios. Such analyses might include: (1) excluding infants who died within 48 h (before intervention completion) to assess survivor bias impact on secondary outcomes; (2) restricting sepsis analysis to culture-proven cases only to eliminate diagnostic ambiguity from clinical sepsis; (3) varying the GA stratification cut-points to test robustness of mortality findings; or (4) propensity score matching to create more balanced comparison groups. These analyses represent important areas for future methodological work and would strengthen confidence in the observed associations prior to definitive RCT evaluation.

## Proposed Next-Step Trial Design: Pragmatic Multicenter RCT

A multicenter pragmatic RCT with individual infant randomization (rather than cluster randomization) should serve as the definitive test of OMC efficacy. We propose co-primary endpoints: (1) NEC incidence and (2) a composite adverse outcome (NEC, culture-proven EOS, or mortality), with randomization stratified by GA category: ≤28 weeks, 29 to 32 weeks, 33 to 36 weeks, given the observed mortality concentration in 29 to 32-week-old infants. The detailed trial design framework is outlined below.

### Study design and setting

A pragmatic, multi-centre, parallel-group, open-label RCT could be conducted across 8 to 12 public-sector Level III NICUs in India. Such a design would enhance generalizability to resource-limited contexts where the burden of preterm mortality and morbidity is greatest. The open-label approach is considered appropriate, as mothers are inherently aware of whether they provide colostrum. Participants would be randomized in a 1:1 ratio to receive oropharyngeal administration of OMC (every 3 h for 48 h, delivered via mother’s fingertip) or to standard care alone. Randomization would be stratified by GA category and centre to minimize confounding and ensure balanced distribution across sites.

### Primary outcomes

The trial should target two coprimary endpoints. First, NEC at Stage 2 or higher as per Bell’s staging criteria documented by 36 weeks postmenstrual age or discharge, whichever comes first. Second, a composite morbidity outcome comprising any of the following: NEC (any stage), culture-positive EOS, or all-cause mortality during hospitalization. This composite approach provides both mechanistic specificity (NEC reduction) and clinical relevance (combined adverse outcome reduction).

### Secondary outcomes

Key secondary endpoints would include EOS incidence (clinical sepsis and culture-proven sepsis separately), days required to achieve full enteral feeds (target 160 mL/kg/day), weight gain velocity (g/kg/day using 2-Point Average Weight model), all-cause mortality stratified by GA stratum, hospital length of stay, and morbidity profile (retinopathy of prematurity, bronchopulmonary dysplasia, intraventricular hemorrhage). Additionally, a substudy would assess immunological biomarkers (serum secretory IgA and lactoferrin concentrations in the first 7 days of life) to validate mechanistic hypotheses, and a parallel health economics analysis would quantify cost-effectiveness and implementation feasibility in resource-limited settings. Long-term neurodevelopmental outcomes at 18 to 24 months corrected age (assessed via modified Bayley Scales-III) would be collected to evaluate whether NEC and sepsis reduction translates to improved developmental trajectories.

### Sample size

Using the observed NEC incidence in this study as the control rate (12%), a sample size of 500 per arm (1000 total participants) would provide 80% statistical power to detect a 40% relative risk reduction (corresponding to an NNT of 14, consistent with the current trial) at a two-sided significance level of 5%, assuming 10% loss to follow-up. An interim futility/efficacy analysis is planned at 50% enrolment (500 participants) to allow early stopping if predefined boundaries are exceeded.

### Key implementation features

Standardized protocols for OMC collection (including optimal timing relative to delivery, container sterility, storage temperature, and expiration timing), storage conditions (room temperature vs refrigeration for time-dependent bioactive component preservation), and administration procedures (frequency, volume, technique, infection control measures) would be mandated across all participating sites. Central data management and outcome adjudication by a masked, independent endpoint review committee would ensure consistency and reduce bias. Primary endpoint assessment (NEC diagnosis) would be verified by a panel of neonatologists blinded to treatment assignment and using standardized diagnostic criteria. An integrated cost-effectiveness analysis would model intervention costs (maternal lactation support, staff training, materials) against cost-benefit of reduced NEC/sepsis treatment expenditure, a critical consideration for implementation decisions in low- and middle-income countries. The immune marker substudy would enroll a random 20% to 30% subset of participants to assess whether observed clinical benefits are accompanied by expected changes in mucosal immune markers (secretory IgA, lactoferrin, pro/anti-inflammatory cytokine ratios).

### Timeline, feasibility, and expected impact

The anticipated trial duration is 3 to 4 years: 18 months for recruitment, 12 months for follow-up, and 6 months for data analysis and manuscript preparation. This timeline is feasible given the high prevalence of preterm births in participating Indian NICUs and established research infrastructure. Results will directly inform clinical practice guidelines for major neonatal societies. If efficacy is confirmed, OMC implementation could prevent 40 to 50 cases of NEC per 1000 VLBW infants in low-income settings, with potential cascading reductions in mortality and long-term morbidity. Furthermore, establishing OMC as an evidence-based, low-cost intervention within routine neonatal practice would align with global efforts to reduce preventable neonatal mortality in low- and middle-income countries and exemplify how pragmatic research in resource-constrained settings can yield scalable solutions.

This proposed RCT design builds directly on the current study’s methodological strengths (clear case definitions, multivariable adjustment, clinical relevance) while addressing inherent observational limitations (non-randomization, temporal bias, survivor bias) through rigorous randomization, multicenter recruitment, central outcome adjudication, and mechanistic validation. The trial represents a natural next step in the evidence continuum and positions the research team and collaborating centers as leaders in advancing neonatal care innovation for vulnerable populations.

## Conclusion

Early oropharyngeal administration of OMC was independently associated with reduced NEC (NNT 14) and EOS (NNT 12) in preterm VLBW infants, with secondary improvements in feeding tolerance and weight gain. These findings suggest potential benefit in a resource-constrained NICU setting.

However, substantial limitations constrain causal inference. The non-randomized observational design with temporal separation between cohorts cannot establish definitive causality despite multivariable adjustment. Additionally, residual confounding from unmeasured variables cannot be excluded. Mortality findings, while numerically favorable in the 29 to 32-week subgroup, represent a secondary outcome and remain hypothesis-generating.

These findings warrant validation through adequately powered multicenter RCTs. If efficacy is confirmed, OMC administration could become a simple, scalable, low-cost intervention to reduce preventable infectious complications in preterm infants, particularly in resource-limited settings where disease burden remains highest yet resources are most constrained. RCT validation is essential before clinical implementation can be recommended.

## Glossary

**1. Colostrum:** Colostrum is yellowish and thick milk secreted during the first 3 to 4 days after birth, when the junctions of the mammary epithelium are open, which allows the translocation of components of the immune system from the maternal circulation to the milk.^8^

**2. Preterm:** Infants born <37 completed weeks of gestation.^3^

**3. Very-low-birth-weight infants:** Birth weight >1000 and <1500 g**.**^3^

**4. Necrotizing enterocolitis:** NEC is an acute inflammatory injury of the distal small and often proximal large intestine. Surgical pathology reveals segmental coagulative necrosis of the mucosa with focal hemorrhage as evidence for ischemia. Other features include intramural gas (pneumatosis) and sloughing of mucosa, submucosa, and muscularis mucosa, which is in contrast to the preserved mucosal integrity in spontaneous intestinal perforation. Universally accepted risk factors include prematurity, bacterial dysbiosis, and formula feeding.^3^

**5. Early-onset sepsis (EOS):** Sepsis occurring in the first 3 days of life is typically caused by organisms transmitted vertically from the mother to the infant before or at the time of birth.^3^

**6. Late-onset sepsis (LOS):** Defined as occurring from 8 to 90 days of life. It can be divided in two distinct entities: disease occurring in otherwise healthy term infants in the community and disease affecting premature infants in NICU. The later is often referred to as hospital-acquired sepsis for premature infants admitted in NICU. For epidemiologic purposes, LOS infections occurring in VLBW infants in the NICU are defined as those occurring at >72 h of life.^3^

## Sources of Funding

This research did not receive any specific grant from funding agencies in the public, commercial, or not-for-profit sectors.

## Ethics Statement and Informed Consent

The study protocol was reviewed and approved by the Institutional Ethics Committee of B.J. Government Medical College and Sassoon General Hospitals, Pune (approval number: BJGMC/IEC/Pharmac/D-0221064-064, dated 22.02.2021). Written informed consent was obtained from parents of prospective cohort participants. A waiver of consent was obtained for retrospective historical data collection.

## Data Availability

The data that support the findings of this study are available from the corresponding author upon reasonable request. Data have been anonymized and are stored securely (password-protected Excel files).

## Author Contributions

Snehal V. Mavchi: conceptualization, data curation, formal analysis, investigation, methodology, validation, writing—original draft, and writing—review and editing; Rahul M. Dawre: conceptualization, methodology, supervision, validation, and writing—review and editing; Isha Deshmukh: formal analysis, investigation, methodology; Sameer Pawar: formal analysis, resources, supervision; Sangeeta Chivale: data curation, methodology, validation; Aarti A. Kinikar: conceptualization, data curation, methodology, supervision, validation, and writing—review and editing.

## Declaration of competing interest

The authors declare that they have no known competing financial interests or personal relationships that could have appeared to influence the work reported in this article.

## References

[1] National Neonatology Forum of India (2023).

[2] National Neonatology Forum of India (2002).

[3] Eichenwald E.C., Hansen A.R., Martin C., Stark A.R. (2017).

[4] Hornik C.P., Fort P., Clark R.H. (2012). Early and late onset sepsis in very-low-birth-weight infants from a large group of neonatal intensive care units. Early Hum Dev.

[5] Claud E.C. (2011). Probiotics and neonatal necrotizing enterocolitis. Anaerobe.

[6] Bocci V. (1991). Absorption of cytokines via oropharyngeal-associated lymphoid tissues. Does an unorthodox route improve the therapeutic index of interferon?. Clin Pharmacokinet.

[7] Ballard O., Morrow A.L. (2013). Human milk composition: nutrients and bioactive factors. Pediatr Clin North Am.

[8] Rodriguez N.A., Vento M., Claud E.C. (2015). Oropharyngeal administration of mother’s colostrum, health outcomes of premature infants: study protocol for a randomized controlled trial. Trials.

[9] Araújo E.D., Gonçalves A.K., Cornetta Mda C. (2005). Evaluation of the secretory immunoglobulin A levels in the colostrum and milk of mothers of term and pre-term newborns. Braz J Infect Dis.

[10] Agarwal R., Sharma N. (2023). Oropharyngeal administration of colostrum as immune support in preterm infants: evidence and practice. Indian Pediatr Rev.

[11] Weigent D.A., Stanton G.J., Johnson H.M. (1983). Interleukin 2 enhances natural killer cell activity through induction of gamma interferon. Infect Immun.

[12] Nasuf A.W.A., Ojha S., Dorling J. (2018). Oropharyngeal colostrum in preventing mortality and morbidity in preterm infants. Cochrane Database Syst Rev.

[13] Sharma D., Kaur A., Farahbakhsh N., Agarwal S. (2020). Role of oropharyngeal administration of colostrum in very low birth weight infants for reducing necrotizing enterocolitis: a randomized controlled trial. Am J Perinatol.

[14] Zhang Y., Ji F., Hu X. (2017). Oropharyngeal colostrum administration in very low birth weight infants: a randomized controlled trial. Pediatr Crit Care Med.

[15] OuYang X., Yang C.Y., Xiu W.L. (2021). Oropharyngeal administration of colostrum for preventing necrotizing enterocolitis and late-onset sepsis in preterm infants with gestational age ≤ 32 weeks: a pilot single-center randomized controlled trial. Int Breastfeed J.

[16] Romero-Maldonado S., Soriano-Becerril D.M., García-May P.K. (2022). Effect of oropharyngeal administration of colostrum in premature newborns ≤32 weeks of gestation on the immune response and neonatal morbidity: a double-blind randomized clinical trial. Front Pediatr.

[17] Patel A.L., Engstrom J.L., Meier P.P. (2009). Calculating postnatal growth velocity in very low birth weight (VLBW) premature infants. J Perinatol.

[18] Carlson S.J., Ziegler E.E. (1998). Nutrient intakes and growth of very low birth weight infants. J Perinatol.

[19] Snyder R., Herdt A., Mejias-Cepeda N. (2017). Early provision of oropharyngeal colostrum leads to sustained breast milk feedings in preterm infants. Pediatr Neonatol.

[20] Seigel J.K., Smith P.B., Ashley P.L. (2013). Early administration of oropharyngeal colostrum to extremely low birth weight infants. Breastfeed Med.

[21] Martín-Álvarez E., Diaz-Castro J., Peña-Caballero M. (2020). Oropharyngeal colostrum positively modulates the inflammatory response in preterm neonates. Nutrients.

[22] Pammi M., Suresh G. (2017). Enteral lactoferrin supplementation for prevention of sepsis and necrotizing enterocolitis in preterm infants. Cochrane Database Syst Rev.

[23] Tao J., Mao J., Yang J. (2020). Effects of oropharyngeal administration of colostrum on the incidence of necrotizing enterocolitis, late-onset sepsis, and death in preterm infants: a meta-analysis of RCTs. Eur J Clin Nutr.

[24] Sudeep K.C., Kumar J., Ray S. (2022). Oral application of colostrum and mother’s own milk in preterm infants—a randomized, controlled trial. Indian J Pediatr.

[25] Huo M., Liu C., Mei H. (2022). Intervention effect of oropharyngeal administration of colostrum in preterm infants: a meta-analysis. Front Pediatr.

